# Shear-induced endothelial mechanotransduction: the interplay between reactive oxygen species (ROS) and nitric oxide (NO) and the pathophysiological implications

**DOI:** 10.1186/1423-0127-21-3

**Published:** 2014-01-13

**Authors:** Hsyue-Jen Hsieh, Ching-Ann Liu, Bin Huang, Anne HH Tseng, Danny Ling Wang

**Affiliations:** 1 Department of Chemical Engineering, National Taiwan University, Taipei 10617, Taiwan; 2 Institute of Biomedical Sciences, Academia Sinica, Taipei 11529, Taiwan; 3 Department of Biomedical Science and Environmental Biology, College of Life Science, Kaohsiung Medical University, Kaohsiung 80708, Taiwan; 4 Institute of Medical Sciences, College of Medicine, Tzu-Chi University, Hualien 97004, Taiwan

**Keywords:** Endothelial cell, Mechanotransduction, Reactive oxygen species (ROS), Nitric oxide (NO), Shear stress, Flow pattern

## Abstract

Hemodynamic shear stress, the blood flow-generated frictional force acting on the vascular endothelial cells, is essential for endothelial homeostasis under normal physiological conditions. Mechanosensors on endothelial cells detect shear stress and transduce it into biochemical signals to trigger vascular adaptive responses. Among the various shear-induced signaling molecules, reactive oxygen species (ROS) and nitric oxide (NO) have been implicated in vascular homeostasis and diseases. In this review, we explore the molecular, cellular, and vascular processes arising from shear-induced signaling (mechanotransduction) with emphasis on the roles of ROS and NO, and also discuss the mechanisms that may lead to excessive vascular remodeling and thus drive pathobiologic processes responsible for atherosclerosis. Current evidence suggests that NADPH oxidase is one of main cellular sources of ROS generation in endothelial cells under flow condition. Flow patterns and magnitude of shear determine the amount of ROS produced by endothelial cells, usually an irregular flow pattern (disturbed or oscillatory) producing higher levels of ROS than a regular flow pattern (steady or pulsatile). ROS production is closely linked to NO generation and elevated levels of ROS lead to low NO bioavailability, as is often observed in endothelial cells exposed to irregular flow. The low NO bioavailability is partly caused by the reaction of ROS with NO to form peroxynitrite, a key molecule which may initiate many pro-atherogenic events. This differential production of ROS and RNS (reactive nitrogen species) under various flow patterns and conditions modulates endothelial gene expression and thus results in differential vascular responses. Moreover, ROS/RNS are able to promote specific post-translational modifications in regulatory proteins (including S-glutathionylation, S-nitrosylation and tyrosine nitration), which constitute chemical signals that are relevant in cardiovascular pathophysiology. Overall, the dynamic interplay between local hemodynamic milieu and the resulting oxidative and S-nitrosative modification of regulatory proteins is important for ensuing vascular homeostasis. Based on available evidence, it is proposed that a regular flow pattern produces lower levels of ROS and higher NO bioavailability, creating an anti-atherogenic environment. On the other hand, an irregular flow pattern results in higher levels of ROS and yet lower NO bioavailability, thus triggering pro-atherogenic effects.

## Review

### Introduction

**Figure 1:**
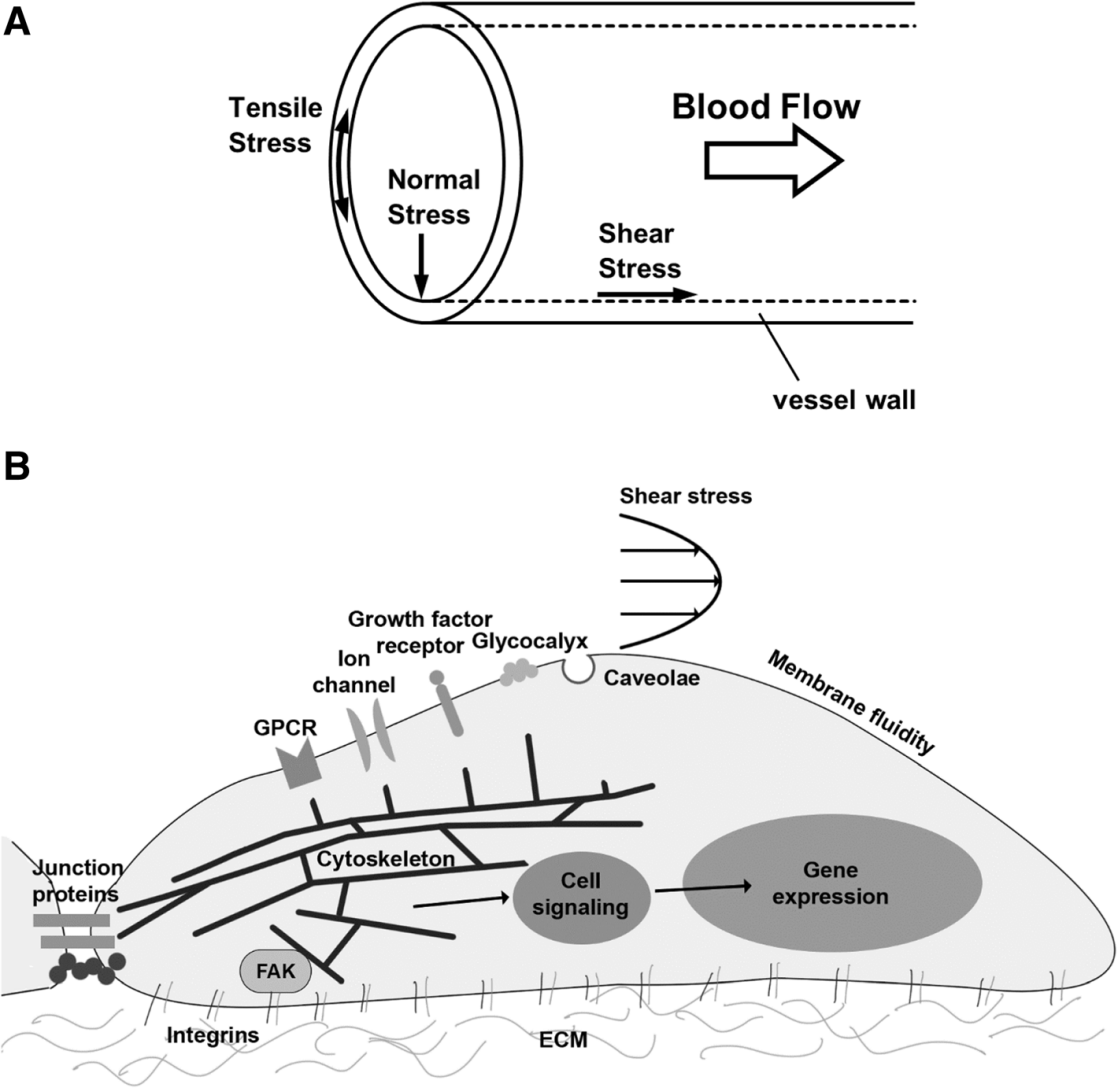
**Hemodynamic forces acting on the blood vessel wall and the potential sensors initiating mechanotransduction. (A)** Hemodynamic forces experienced by the blood vessel wall including: 1) shear stress, which is the tangential frictional force acting on the vessel wall due to blood flow, defined as force/wall area (e.g., dyn/cm^2^); 2) normal stress, which is the force acting perpendicularly on the vessel wall due to hydrostatic pressure; and 3) tensile stress, which is the force acting on the vessel wall in the circumferential direction due to stretch of the vessel wall. **(B)** Potential mechano-sensors likely to initiate mechanotransduction in endothelial cells, including G protein-coupled receptor (GPCR), mechano-activated ion channels, growth factor receptor, glycocalyx, caveolae, membrane lipids (fluidity), junction proteins, cytoskeleton network, integrins, focal adhesion kinase (FAK), etc. [5]. In mechanotransduction process the mechanical signals trigger the perturbation of these mechano-sensors, thus generating biochemical signals and initiating mechano-sensitive signaling cascades that lead to downstream gene expression.

Blood vessels are constantly under the influence of hemodynamic forces including: 1) shear stress, which is the tangential frictional force acting on the vessel wall due to blood flow, defined as force/wall area (e.g., dyn/cm^2^); 2) hydrostatic pressure, the perpendicular force acting on the vascular wall; and 3) cyclic strain, the circumferential stretch of the vessel wall (Figure 1A) [1]. As an interface between the blood flow and vessel wall, endothelial cells (ECs) is exposed to these hemodynamic forces. Indeed, it is well established that the signaling arising from EC-blood flow interaction are important determinants of vascular homeostasis. ECs and neighboring smooth muscle cells (SMC) are also involved in signaling communication, the net result of which influences vascular remodeling, myogenic tone and vascular response to vasoactive agonists.

Extensive studies over the past few decades have showed that vascular ECs sense mechanical force and transduce them into biological responses [2-5], termed as mechanotransduction. This complex process involves perturbation of sensors that generate biochemical signals that initiate complex and multiple signaling cascades that eventually drive short- and long- term vascular responses. Candidate sensors are ion channels, receptor tyrosine kinases, G protein-coupled receptors, junction proteins, integrins, cytoskeletal network, membrane lipids and the glycocalyx (Figure 1B) [5].

The geometric structure of the vascular tree comprises straight, curved, branched, converged, diverged, and other complex features, thus rendering the hemodynamic environment in the vascular tree extremely complicated. In the straight part of an artery, the hemodynamic flow pattern is typically laminar with an average shear stress of 10–70 dyn/cm^2^ on the vascular ECs, and thus the flow condition is termed regular flow. However, in the curved, branched, and diverged regions of arterial tree, the hemodynamic flow becomes disturbed, leading to the formation of eddies, and the occurrence of low and reciprocating (oscillatory) shear stress regions, and thus the flow condition is termed irregular flow [1]. *In vivo* observations have revealed that atherosclerotic lesions preferentially localize at bends and bifurcations in the arterial tree where irregular flow is likely to occur; it is now well accepted that regular flow maintains vascular homeostasis while irregular flow lead to unfavorable vascular responses that eventually result in vascular diseases [6]. Later studies have shown that regular flow (either steady or pulsatile) causes activation and regulation of anti-inflammation and anti-atherogenic genes, whereas irregular flow with a low, reciprocating (oscillatory) shear stress, or disturbed flow pattern increases transcription of pro-atherogenic genes [1].

Studies of the past decade indicate that reactive oxygen species (ROS) generated in response to altered flow or cyclic strain settings play a key role in the signaling mechanisms and affect vascular homeostasis [7-9]. ROS (a collective term that refers to oxygen radicals such as superoxide, O_2_^˙-^ and hydroxyl radical, OH. and to non-radical derivatives of O_2_, including H_2_O_2_ and ozone (O_3_) in cells and tissue is determined not merely by cellular production but also by the antioxidant defenses; indeed antioxidant enzymes such as superoxide dismutase, catalase, glutathione peroxidase, thioredoxin, peroxiredoxins and heme oxygenase-1 regulate and often reduce the level of ROS in biological systems.

Apart from ROS, reactive nitrogen species [RNS such as nitric oxide (NO), nitrogen dioxide (NO_2_^-^), peroxynitrite (OONO^-^), dinitrogen trioxide (N_2_O_3_), nitrous acid (HNO_2_), etc.] also play a complex role in endothelial disorders. Nitric oxide (NO) (produced from sources such as endothelial nitric oxide synthase) released from the endothelium due to stimuli such as shear stress, regulates the vascular environment by inhibiting the activity of proinflammatory agents (cytokines, cell adhesion molecules and growth factors released from endothelial cells of the vessel wall and from platelets on the endothelial surface). The interaction of NO with ROS causes the production of several RNS that potentiate cellular damage. This does not generally occur under normal cellular conditions, where the limited ROS and NO produced contribute to vascular homeostasis. However under conditions of excessive ROS production i.e. oxidative stress, elevated levels of ROS cause a decrease in bioavailability of NO in addition to production of RNS such as peroxynitrite that are implicated in oxidative and nitrosative damage [10,11]. NO, besides its direct role in vascular function, also participates in redox signaling by modifying proteins (via S-nitrosation of cysteine residue) and lipids (via nitration of fatty acid) [12,13].

**Figure 2:**
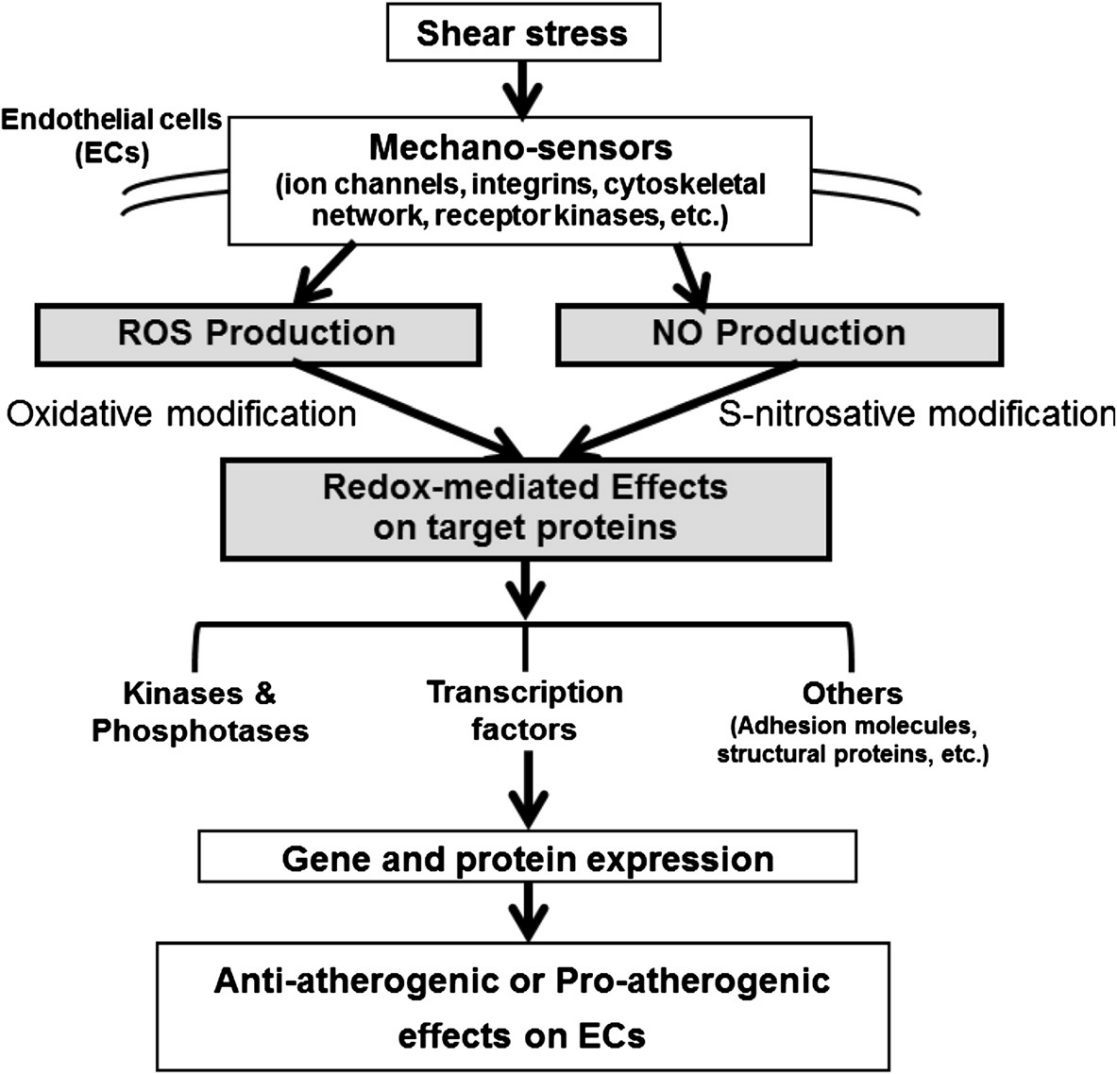
**Roles of ROS/NO in mechano-sensor mediated redox signaling in ECs exposed to shear stress.** Hemodynamic shear stress is detected by various mechano-sensors present on the membrane of ECs, triggering a network of signaling pathways that alter gene and protein expression, eventually leading to anti-atherogenic or pro-atherogenic effects on ECs. In this process, ROS triggers oxidative modification and NO triggers S-nitrosation of many target molecules, together with activation of antioxidant and pro-oxidant enzymes to regulate the redox status of ECs. Shear stress with a regular flow pattern (steady or pulsatile) produces lower levels of ROS (hence to be anti-atherogenic) than shear stress with an irregular flow pattern (disturbed or oscillatory) that is pro-atherogenic.

Research of the past decade has documented that overproduction of ROS and/or deregulation of RNS production drives development of heart and cardiovascular diseases [10,11,14-17]. The present review emphasizes the interplay between ROS and NO in the context of shear stress-induced mechanosignaling. Our current concepts based on ample published evidence and summarized in Figure 2 are as follows: 1) hemodynamic shear stress sensed by various mechanosensors on vascular ECs, trigger signaling pathways that alter gene and protein expression, eventually giving rise to anti-atherogenic or pro-atherogenic responses in the vascular wall depending on the flow patterns. 2) These signaling pathways are ROS/RNS mediated and the eventual physiological responses depend on a large part on the interactions between ROS and NO and these interactions-modulating redox signalings that drive physiological or pathological processes. The following sections will discuss the shear signaling initiated by various flow patterns, and the effect of ROS/NO interactions on redox signaling in the vasculature.

### Sources of ROS and NO production in response to shear

In general, potential sources of ROS production in ECs include NADPH oxidase (Nox), xanthine oxidase, mitochondria and uncoupled eNOS. In most vascular beds under normal physiological conditions, Nox oxidases appear to be the predominant sources of ROS in ECs under shear stress. Shear stress exerted by blood flow to ECs is sensed via above-mentioned mechano-sensors on EC. These initiate a complex signal-transduction cascade which produces ROS and NO. NO is generated by eNOS activation in which shear stress plays widely regulatory roles at the transcriptional, posttranscriptional and posttranscriptional levels.

### NAD(P)H oxidase (Nox)

NADPH oxidase (Nox) upon activation uses NADPH to reduce oxygen to superoxide anion. Activation of this enzyme requires the assembly of Nox (1–5), regulatory subunits (p22phox, p47phox, p67phox, p40phox) and the small GTPase Rac. Among Nox homologs (Nox 1–5 and Duox 1–2) [17], only Nox 1, 2, 4 and 5 enzymes are known to express in the vascular system. Nox2, also known as pgp91, was first known as an enzymatic complex responsible for respiration burst in phagocyte. Human umbilical vein ECs in culture express all of the components of traditional phagocytic NADPH oxidase (including Nox2). Nox1, similar to Nox2, forms a membrane-bound cytochrome with p22phox. However, p47phox and p67phox can be replaced by Noxo1 and Noxa1. Compared with Nox2, Nox1 possesses moderate physiological activity due to its low expression and specific regulatory units and signaling cascades. Nox4 is frequently coexpressed with Nox1 and Nox2. Similar to Nox1 and Nox2, Nox4 binds to p22phox. However, Rac1 does not activate Nox4. Nox4 mRNA level in ECs is significantly higher than Nox1 and Nox2 and is indicated to be a primary source of intracellular ROS in ECs. Nox5 expression is limited to fewer tissues including VSMCs and ECs. The striking structural difference of Nox5 from other Nox enzymes is the presence of an additional cytosolic N-terminal segment, containing 4 calcium binding EF-hands. An increase of intracellular calcium concentration in ECs triggers high superoxide production by Nox5. Under physiological conditions Nox proteins and their products superoxide and hydrogen peroxide act as structural and signaling molecules to regulate cell growth and differentiation, wound repair and control of vascular tone. In this review, we emphasize on the role of Nox enzymes in shear stress-induced ROS production.

**Figure 3:**
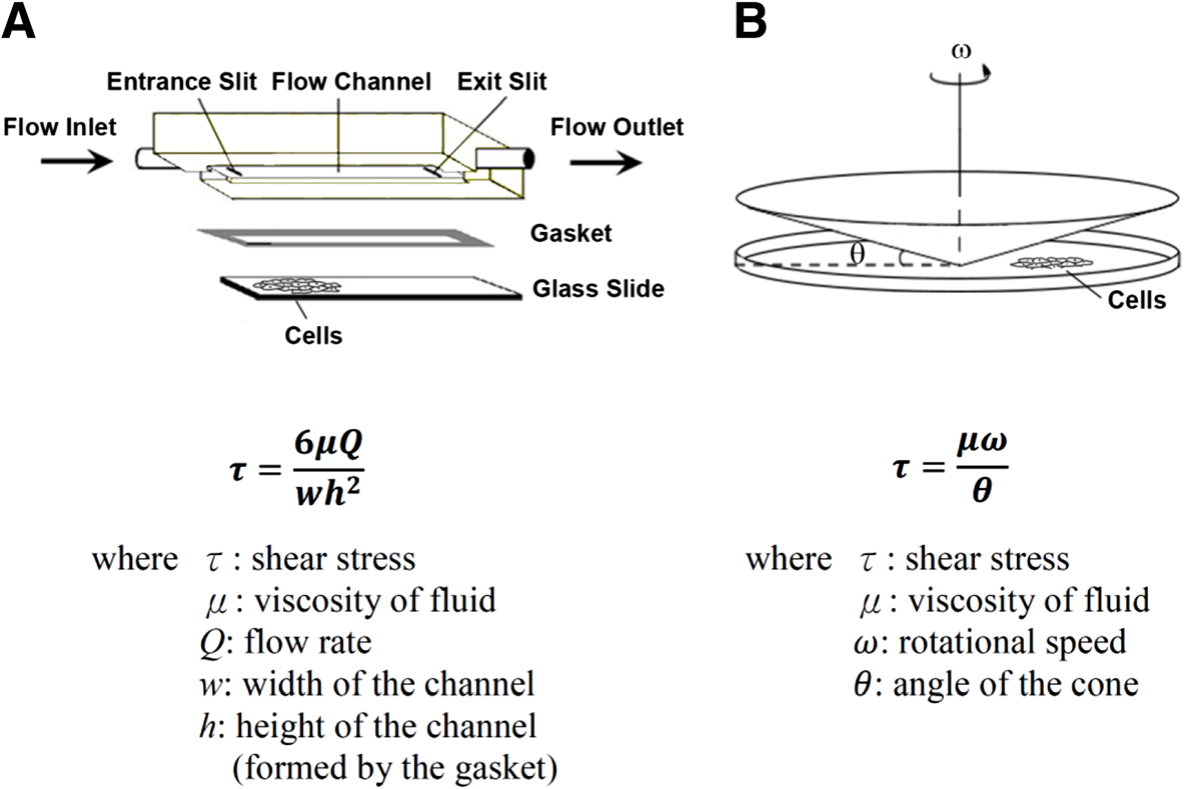
**Devices used to carry out in vitro studies to examine the influence of flow (shear stress) on ECs. (A)** Parallel-plate flow chamber. In a parallel-plate flow chamber system ECs monolayers are exposed to well-defined flow and thus shear stress (denoted by τ) in a small channel with fixed height. **(B)** Cone-and-plate flow chamber. In a cone-and-plate flow chamber system ECs monolayers are exposed to shear stress (τ) generated by a rotating cone. The magnitude of shear stress can be calculated using the respective formula shown in **A** and **B**.

**Figure 4:**
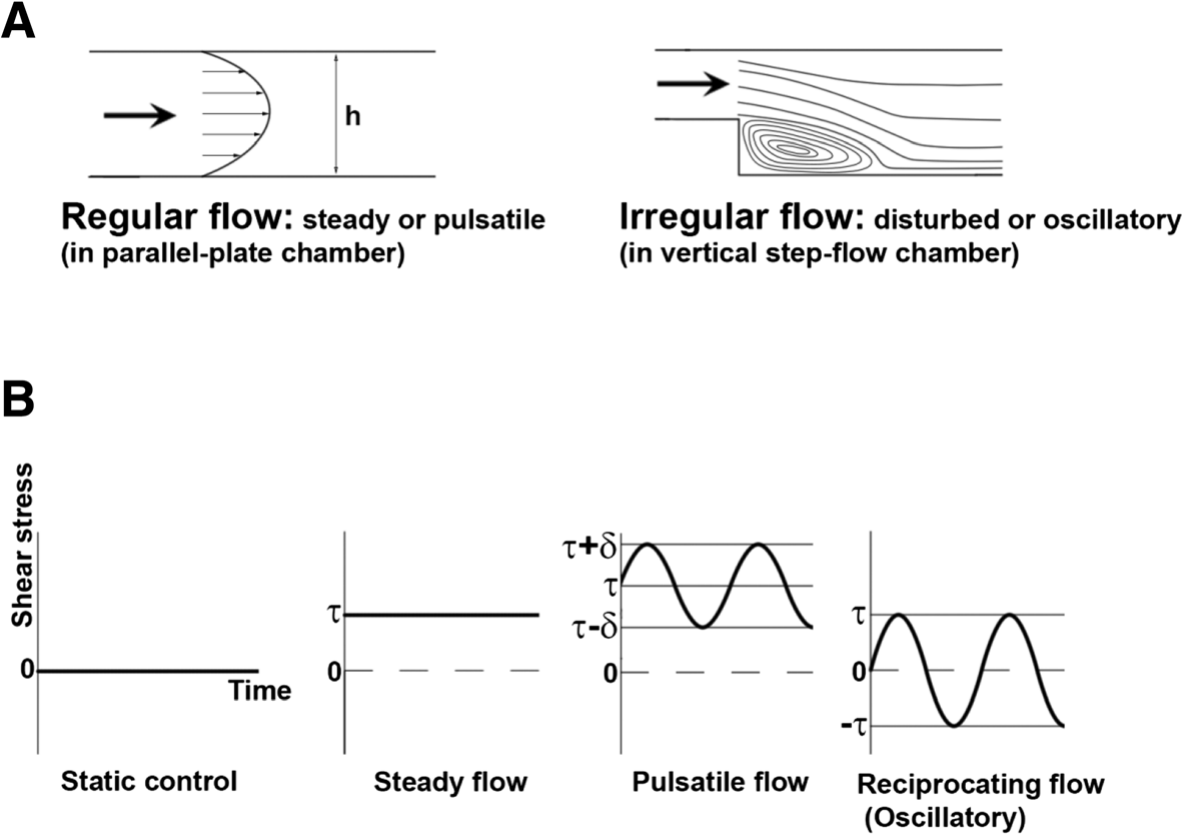
**Classification and description of flow patterns. (A)** Illustration of regular flow and irregular flow. The flow pattern in a parallel-plate flow chamber is laminar with a parabolic-like velocity profile and the flow condition is termed regular flow, which can be steady or pulsatile. In contrast, the flow pattern in a vertical step-flow chamber is disturbed with the formation of eddies and separation of streamlines and thus the flow condition is termed irregular flow, which can be disturbed or oscillatory. **(B)** Demonstration of various types of flow. According to the magnitude of shear stress and variation of shear stress with time, they can be categorized as static control, steady flow, pulsatile flow, and reciprocating (oscillatory) flow. For static control, no shear stress is produced because there is no flow. For steady flow, a physiological level of shear stress (τ) is produced by the flow. For pulsatile flow and reciprocating (oscillatory) flow, cyclic change (e.g. 1 Hz) in the level of shear stress is maintained, but the average level of shear stress (τ) of pulsatile flow is relatively higher in comparison with reciprocating (oscillatory) flow, for which the average level of shear stress is zero or very low.

To study the shear flow effects on ECs, various apparatus have been designed to examine the influence of various flow patterns (and thus shear stress) on ECs in vitro (Figures 3 and 4 illustrate the detailed design and provide the description of these apparatuses and types of flow). Using these chambers, it was observed that steady laminar flow (5 dyn/cm^2^) led to only a transient induction of Nox activity [18,19]; in contrast, atherogenic oscillatory shear stress (OSS, ± 3 ~ 5 dyn/cm^2^) or negative shear stress (flow reversal) caused sustained Nox activity and O_2_^˙-^ production [20,21], indicating a role for directional activation of Nox. However, prolonged shear stress (30 dyn/cm^2^, for 24 h) was observed to down-regulate Nox subunits p47phox and Nox2 (gp91phox); O_2_^˙-^ production was also reduced [22]. Similarly, ECs exposed to long-term arterial laminar shear stress decreased Nox4 expression and reduced O_2_^˙-^ production [23]. The Nox4 promoter contains an antioxidant response element (for Nrf2 binding) and an Oct-1 binding site that are responsible for flow-dependent down-regulation of Nox4 [23]. However, OSS upregulates Nox 1 and Nox 2 mRNAs while suppressing or inducing Nox4 [24,25]. From knockdown experiments it seems that OSS-induced ROS was derived from Nox1 [25]. Pulsatile shear stress (PSS, mean shear stress of 25 dyn/cm^2^) downregulates Nox2 and Nox4 mRNAs [24]. The effect of flow and various flow patterns on Nox5 or Duox activity has not been investigated yet. But expression and activity of Noxes 1, 2 and 4 are differentially regulated by the flow pattern that contributes to ROS production in ECs.

### Mitochondrial respiratory chain, xanthine oxidase and uncoupled eNOS

Oxidative phosphorylation in the mitochondria causes the proton translocation across the mitochondrial inner membrane to intermembrane space, generating an electrochemical proton gradient that is expressed as mitochondrial membrane potential (ΔΨ_m_) and mtROS level increases exponentially as ΔΨ_m_ is hyperpolarized above −140 mV. Previous studies showed that cyclic strain induced ROS production and mitochondria was the source of ROS [26]. With an acute induction of shear stress, mitochondrial complexes I and III generate ROS in coronary arteries [27]. Oscillatory flow was shown to induce mitochondrial superoxide production via NADPH oxidase-JNK signaling pathway [21]. Steady laminar shear-induced NO production mediates a sustained suppression of the activities of respiratory complexes I, II/III, and IV [28]. Mitochondrial ROS generation is regulated by shear stress due to the eNOS-derived NO and RNS inhibit mitochondrial electron transport [28]. Shear stress thus has antioxidant effects in ECs as it partly suppresses mitochondrial respiration via NO. Xanthine oxidase (XO) utilizes NADH, O_2_ and xanthine/hypoxanthine to generate O_2_^˙-^ and H_2_O_2_. Increased XO activity reportedly impairs flow-dependent and endothelium-dependent vasodilation [15,16,29]. Under oscillatory flow, endothelial ROS production in ECs is reported to be derived mainly from XO [30]. Under conditions of limiting L-arginine or cofactor tetrahydrobiopterin (BH4), eNOS is able to exhibit NADPH oxidase activity (eNOS uncoupling), and the resulting O_2_^˙-^ may contribute to vascular dysfunction. Endothelial dysfunction in various pathological settings exhibits eNOS uncoupling [31]. Nox1 activation and upregulation mediate eNOS uncoupling in diabetes patients [32] and in endothelium-dependent relaxation impairment [33]. Shear stress-induced NO levels are significantly lower in vessels of aged rats, and this is associated with increased O_2_^˙-^ production from eNOS uncoupling [34].

### Influence of shear stress on endothelial nitric oxide oxidase (eNOS)

Endothelial eNOS is a constitutively expressed enzyme, it is also regulated at the transcriptional, posttranscriptional and posttranslational levels [35,36]. Shear stress can activate eNOS by several signaling pathways. Studies on the onset of shear indicates that ECs rapidly respond to shear stress with an acute but transient increase in intracellular calcium that enhances the calmodulin binding to eNOS and increases eNOS activity [37]. In addition, calmodulin activates calmodulin kinase II to phosphorylate eNOS on S1177/1179. However, an increase in diacylglycerol levels can activate PKC to phosphorylate T497 but negatively regulates eNOS activity. Shear stress, similar to VEGF, estrogen and bradykinin, can activate G proteins that stimulate PI3K/Akt [38] and adenylate cyclase [39,40], both of which lead to phosphorylation of serine residues (S617 and S1177/1179 by Akt, S635 and S1177/1179 by PKA) on eNOS and hence its activation [36].

Graded increase in shear promotes eNOS expression and activity. Li et al. using artificial capillary modules to study the effects of pulsatile flow/shear stress on ECs reported that ECs adapted to low physiological flow (3 dyn/cm^2^) followed by high shear (10, 15, 25 dyn/cm^2^) environments for up to 24 h showed graded elevation of eNOS mRNA, protein expression and NO release [41]. In addition to the rapid PI3K-dependent eNOS phosphorylation on S1177, acute shear exposure reduced phosphorylation at T495 due to a decrease in PKCδ activity [41,42]. However, a prolonged NO production requires an increase of eNOS expression and enzyme activation. Furthermore, ECs with catalase overexpression attenuated the acute shear-induced phosphor-S1177 eNOS and NO production, confirming that acute shear-mediated increase in ROS plays a role in the acute eNOS activation. Under prolonged shear stress, PI3K pathway is not involved in the increased eNOS expression.

Studies with flow chamber module demonstrated that laminar flow triggered AMP-activated protein kinase (AMPK) activation and subsequent phosphorylation of eNOS at S635 and S1179 [43,44]. Recent studies further showed that SIRT1, an NAD^+^-dependent class III histone deacetylase, played a role by deacetylating eNOS at Lys496 and 506 in calmodulin-binding domain of eNOS and thereby increased eNOS activity [45]. Further studies by Chen et al. demonstrated that shear stress increased SIRT1 level and activity and SIRT1 level was higher in ECs exposed to physiologically relevant pulsatile flow than those under pathologically relevant oscillatory flow. They further showed that AMPK phosphorylation of eNOS was required for the SIRT1 deacetylation of eNOS [46]. Thus, atheroprotective flow increases the level of SIRT1, and SIRT1 acts together with AMPK to promote NO production in endothelium.

Fluid shear stress also induces transcriptional factors, such as Krüppel-like factor (KLF2), which upregulates eNOS expression [47-49]. Steady or PSS markedly activates Nrf2 and induces Nrf2-regulated antioxidant genes, such as heme oxygenase-1 (HO-1) and thioredoxin reductase-1 (TrxR1), and this reduces the level of intracellular O_2_^˙-^, thereby increasing the level of bioavailability NO [50-52]. Thus, ECs under steady or physiological PSS have reduced intracellular ROS and increased bioavailability of NO.

### Flow patterns and the production of ROS and NO

As mentioned above, the geometric structure of the vascular tree drives changes in blood flow which may cause endothelial dysfunction. To carry out in vitro study to examine the influence of flow on ECs, a parallel-plate flow chamber system has been designed for the exposure of ECs monolayers to well-defined flow (and thus shear stress) in a small channel with fixed height (Figure 3A) [53]. Another in vitro system commonly used for this purpose is the cone-and-plate flow chamber system, in which ECs monolayers are exposed to shear stress generated by a rotating cone (Figure 3B) [1]. Figure 4A illustrates the flow pattern of regular flow (which can be steady or pulsatile) created in a parallel-plate flow chamber, and also the flow pattern of irregular flow (which can be disturbed or oscillatory) created in a vertical step-flow chamber [1]. Figure 4B demonstrates various types of flow. According to the magnitude of shear stress and variation of shear stress with time, they can be categorized as static control, steady flow, pulsatile flow, and reciprocating (oscillatory) flow (Figure 4). Our group used the parallel-plate flow chamber system to investigate the effects of laminar flow on the ROS levels and ROS-related signaling in ECs. Here we briefly discuss the differential influence of regular flow vs. irregular flow on the production of ROS and NO, which may contribute to the anti-atherogenic or pro-atherogenic effects.

### Effect of steady or pulsatile flow (regular flow)

We and others have demonstrated that ECs exposed to steady or pulsatile flow with normal shear stress (regular flow) increased intracellular levels of ROS that enhanced the expression of Nrf2, KLF2, c-*fos*, superoxide dismutase (SOD), HO-1, and intracellular adhesion molecule-1 (ICAM-1) [19,48,54-56].

**Figure 5:**
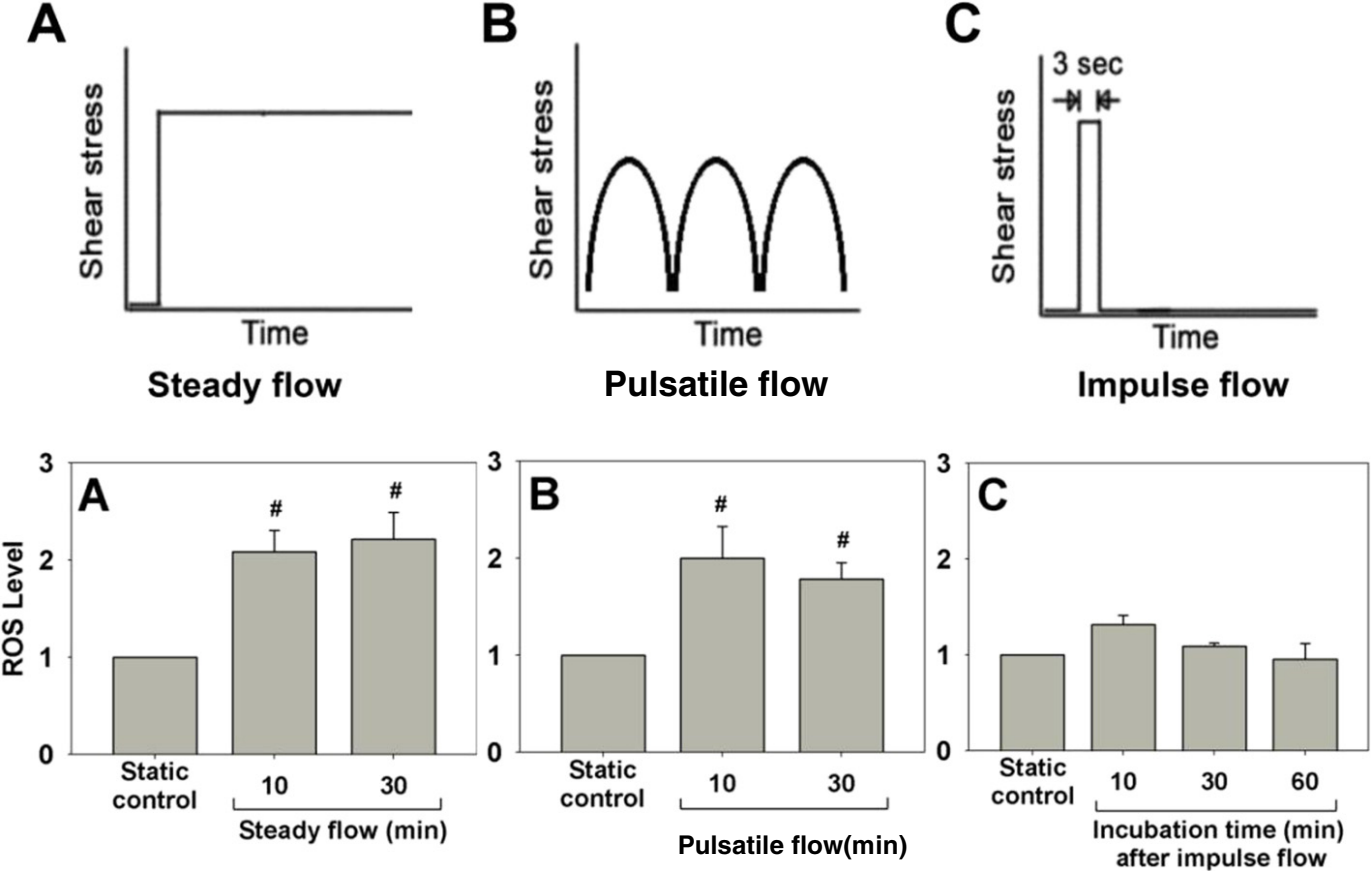
**Relative levels of ROS in ECs exposed to various flow patterns. (A)** Steady flow (step shear stress increase from 0 to 13.5 dyn/cm^2^ and then maintained for 10 or 30 min), **(B)** Pulsatile flow (periodic variation in shear stress from 3 to 25 dyn/cm^2^, 1 Hz), **(C)** Impulse flow (step increase in shear stress from 0 to 13.5 dyn/cm^2^ for 3 seconds). ROS levels in ECs exposed to various flow patterns were determined by measuring the 6-carboxy-DCF (an ROS probe) fluorescence and normalized to the static control. Data represent the means ± S.E. of three experiments. # P <0.05 vs. static control. (Yu-Chih Tsai, Master’s Thesis, Department of Chemical Engineering, National Taiwan University, 2002).

ECs exposed to shear stress of 20 dyn/cm^2^ had increased intracellular O_2_^˙-^[56]. Furthermore, laminar flow with shear stress of 15, 25, or 40 dyn/cm^2^ for 15 min led to a 0.5- to 1.5-fold increase of intracellular ROS [19]. A concomitant increase in antioxidant activity in ECs along with ROS generation was also noticed [19]. We have also demonstrated that steady flow and pulsatile flow led to a 1-fold increase of ROS, but impulse flow triggered a small and transient increase of ROS (Figure 5). These results suggest that different flow patterns may induce ROS production to a different extent.

It has been reported that a steady or PSS produces less O_2_^˙-^ than an OSS [18,57]. A steady high shear stress to ECs consistently suppressed ROS levels more than low shear stress [58]. ECs subjected to a prolonged laminar shear stress (30 or 75 dyn/cm^2^) for 24 h reduced O_2_^˙-^ formation and ROS levels [23,59]. Recent study using a hemodynamic Lab-on-a-chip system, however, showed no significant increase of ROS when ECs under constant shear stress (30 dyn/cm^2^), in contrast to the sustained increase of ROS level in ECs under physiological conditions of PSS [60]. Thus, these data are inconsistent with respect to ROS levels in ECs exposed to different flow patterns or conditions. The inconsistencies could be due to different methods used to measure ROS, prompting Dikalov et al. to recommend the use of two methods for ROS measurement [61]. In addition, different sources of ECs (vein or artery; human or bovine), different flow systems, or minor differences in cell culture and serum-starvation conditions could also be the factors contributing to these inconsistencies, as reported [62]. Moreover, the duration of flow is another factor that can affect the ROS levels. Long term flow appears to down-regulate ROS through antioxidant response mediated by antioxidant enzymes such as superoxide dismutase (SOD), catalase, glutathione peroxidase, thioredoxin, peroxiredoxins and HO-1. Despite these discrepancies, it is generally accepted that ROS become moderately elevated in ECs exposed to regular flow but that prolonged exposure to regular flow is mainly associated with an antioxidant response, unless the shear stress is abnormally high [63]. The moderately elevated ROS may act as messenger molecules in vascular adaptation to hemodynamic perturbation and thus play important roles in vascular physiology.

On the other hand, NO plays important roles in vasodilation and anti-inflammation. Many studies have examined the effects of different flow types on NO production in ECs. Frangos *et al.* investigated NO production in ECs exposed to three types of flow: 1) step flow, a sudden increase of shear stress from 0 to 20 dyn/cm^2^ and maintenance at 20 dyn/cm^2^; 2) ramp flow, a gradual increase in shear stress from 0 to 20 dyn/cm^2^ and maintenance at 20 dyn/cm^2^; 3) impulse flow, a 3-second pulse of 20 dyn/cm^2^. Their results indicated that NO production occurs by two independent pathways. Step flow and impulse flow induced a transient burst of NO production that is G protein-dependent, and step flow and ramp flow induced sustained NO production that is G protein-independent. It is noteworthy that step flow, which contains both a rapid increase and a steady flow component, stimulates both pathways [64]. In general, NO production in ECs is continuously elevated by regular flow. NO may modify proteins and lipids and also regulate transcriptional factors and adhesion molecules expression in the vasculature. In addition, NO may react with ROS to form peroxynitrite that modulates various cellular events. However, these peroxynitrite-induced effects are limited under regular flow condition, since regular flow results in only a moderate elevation in ROS production. Even though a continuous NO production is present, the amount of peroxynitrite (and hence its influence) is quite restricted.

### Effect of disturbed or oscillatory flow (irregular flow)

**Figure 6:**
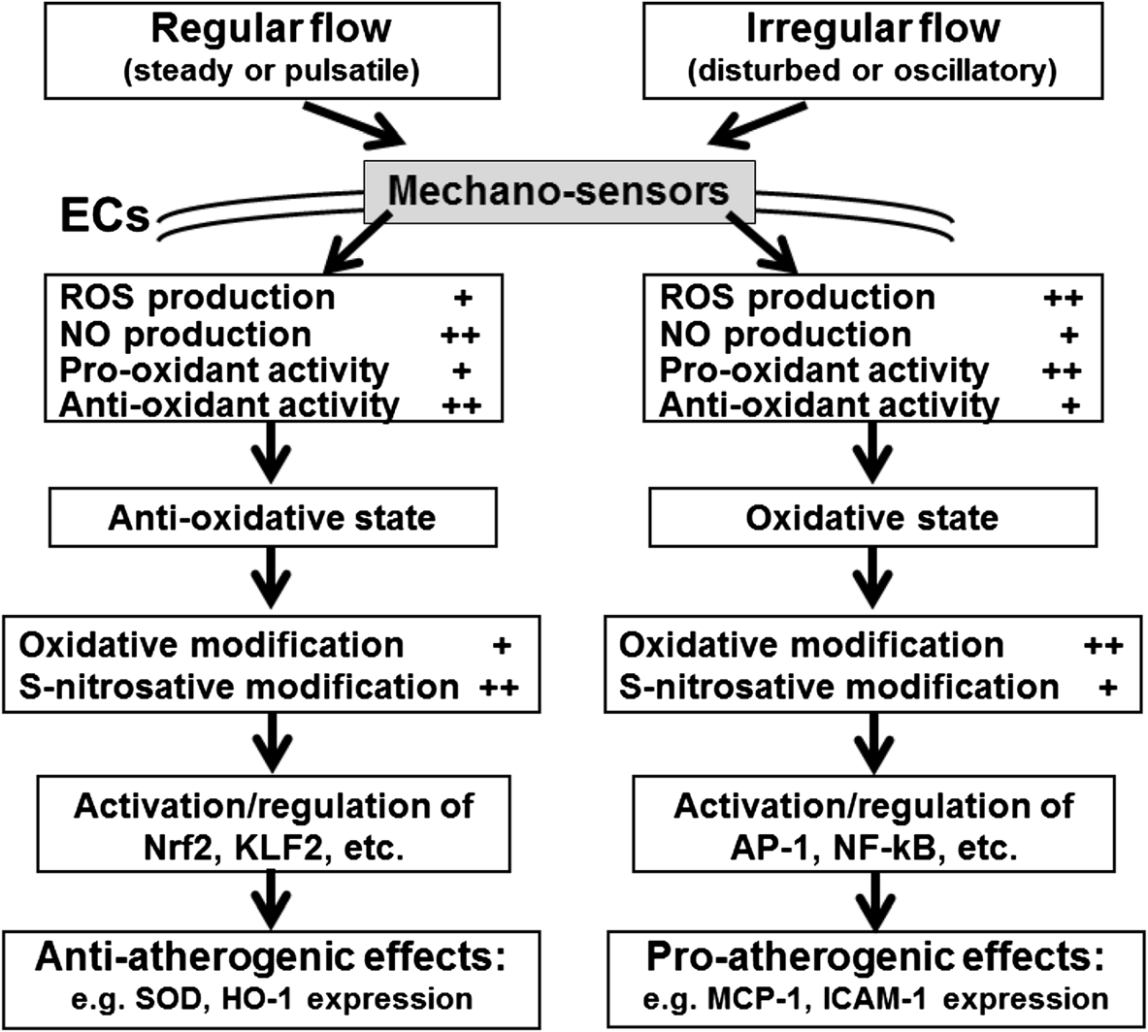
**Pro- or anti- atherogenic effect of flow patterns through different redox signalings and genes expression.** A regular flow pattern (steady or pulsatile) produces lower levels of ROS and pro-oxidant activity, yet higher NO bioavailability and anti-oxidant activity, that result in an anti-oxidative state, favoring the activation/regulation of key transcription factors such as Nrf2, KLF2 to promote anti-atherogenic environment by enhancing the expression of SOD, HO-1, etc. On the other hand, an irregular flow pattern (disturbed or oscillatory) produces higher levels of ROS and pro-oxidant activity, yet lower NO bioavailability and anti-oxidant activity, that result in an oxidative state, favoring the activation/regulation of key transcription factors such as AP-1, NF-κB for pro-atherogenic environment by enhancing the expression of MCP-1, ICAM-1, etc. ++: relatively higher; +: relatively lower.

As mentioned, earlier clinical evidence indeed points out that atherosclerotic lesions preferentially emerge at arterial bifurcations and curvatures, where irregular flow is usually happen [1,63,65]. The effect of disturbed or oscillatory flow (irregular flow) on NO production in ECs has been investigated recently. An *ex vivo* preparation of porcine arteries exposed to the flow of a physiological solution through the vessels in the forward and reverse directions (oscillatory flow) indicated that NO concentration was significantly lower during reverse flow [66]. Furthermore, addition of a O_2_^˙-^ scavenger returned the NO concentration during reverse flow to that of forward flow. This suggests that flow reversal has a pro-atherogenic effect that may be associated with increased O_2_^˙-^ production [66]. A study comparing the effects of oscillatory flow with a mean stress of 0.02 dyn/cm^2^ and pulsatile flow with a mean stress of 23 dyn/cm^2^ on ECs indicated that oscillatory flow significantly upregulated Nox4 (an NADPH oxidase subunit) and increased O_2_^˙-^ production. In contrast, pulsatile flow upregulated eNOS expression and increased NO production [67]. These results suggest that an imbalance in O_2_^˙-^ and NO under oscillatory flow leads to the formation of peroxynitrite, a key molecule which may trigger many pro-atherogenic events [67]. Elsewhere studies also showed altered shear triggers membrane depolarization for PI3K/Akt activation to produce ROS [68]. Taken together, the aforementioned studies suggest that shear stress with a regular flow pattern produces lower levels of ROS and more bioavailable NO (thus to be anti-atherogenic). In contrast, shear stress with an irregular flow pattern generates higher levels of ROS and less available NO that results in pro-atherogenic effects, as described in Figure 6.

### Influence of shear stress on ROS/NO redox signaling and downstream events

An important feature underlying redox signaling is the reversible (covalently oxidative or nitrosative) modification of specific cysteine (Cys) thiol residues that reside within active and allosteric sites of proteins, which results in alternation of protein functions. These Redox-sensitive thiols play an essential role in cellular redox signalings and are thus associated with homeostatic maintenance. S-nitrosative modification occurs by means of oxidative reaction between NO and Cys thiol in the presence of an electron acceptor or through transnitrosylation from S-nitrosothiol to another Cys thiol. The oxidation or nitrosation of redox thiol is determined by the relative fluxes of ROS and NO and the proximity of the thiol-protein to the sources of ROS or NO generation. Thus, different ROS and NO production rates by various flow patterns and the subsequent ROS/RNS interplay resulting in oxidative or nitrosative modification of thiol-containing molecules can have profound effects on the signaling cascades and downstream events.

The numerous signaling pathways that are activated by flow feature ROS and NO as important regulators of redox signaling. The effects of shear-induced ROS/NO on redox signaling and downstream events are categorized into four aspects including kinases/phosphatase, transcriptional factors, adhesion molecules, and protein-modifications.

### Effect of shear-induced ROS/NO on kinases and phosphatases

**Figure 7:**
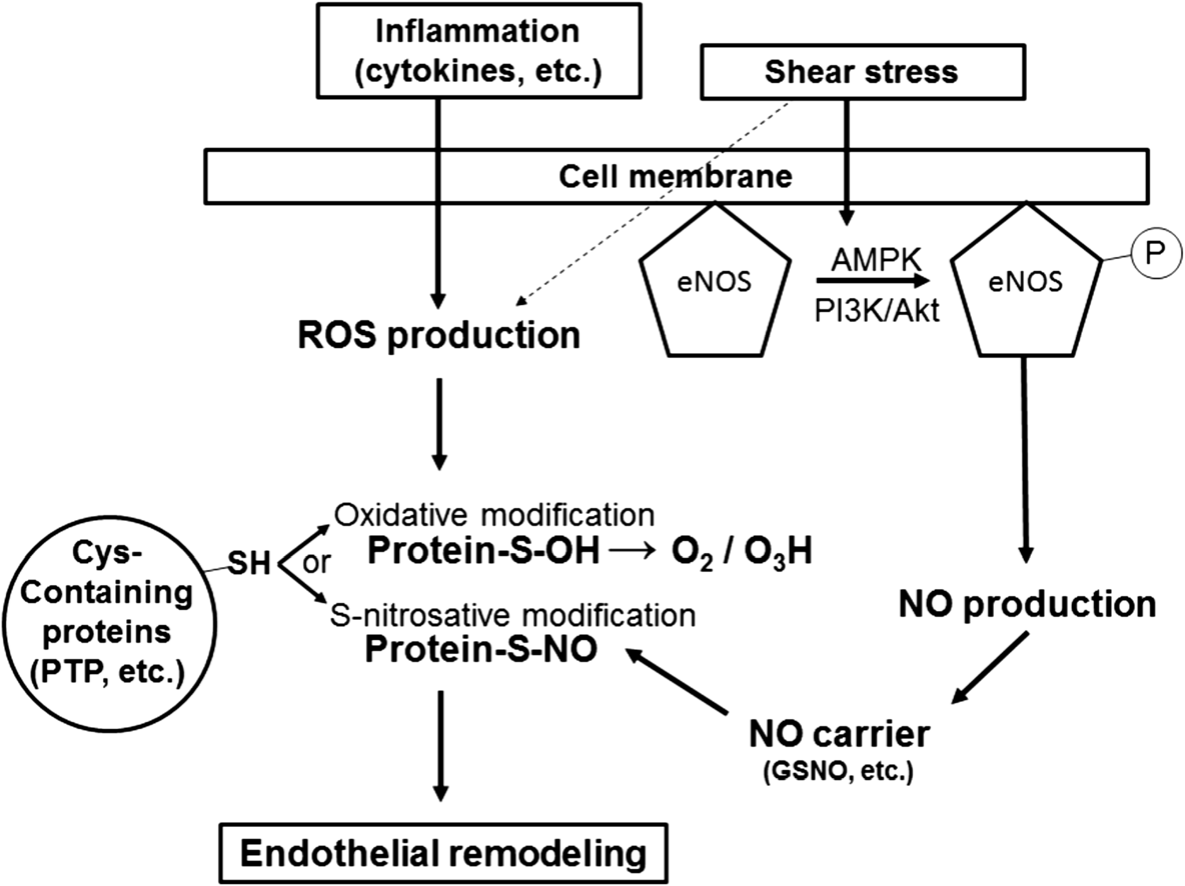
**Model of the effect of shear stress on S-nitrosation of redox-sensitive Cys-containing proteins in ECs.** Shear stress activates endothelial nitric oxide synthase (eNOS), leading to an increased level of NO and increased S-nitrosation (S-NO) of proteins via NO carrier proteins or peptides. Shear stress-induced protein’s S-nitrosation may prevent the irreversible oxidative modification of proteins (S-OH and S-O_2_/O_3_H) that otherwise would occur during severe inflammation [73].

Endogenous ROS and reactive nitrogen species (RNS) can act reversibly by altering functions of various target kinases/phosphatases. Increased activation of protein kinases such as Src, PI3K, MAPK, PKA, PKG and PKC was demonstrated by the thiol oxidation [31]. In contrast, oxidative modification of phosphatases such as PTEN and MAPK phosphatase suppresses their activities [31]. It is conceivable that laminar shear stress-induced ROS suppresses PTEN and MAPK phosphatase thus increasing the activation of protein kinases. Similarly, NO-mediated S-nitrosation of redox thiol in protein kinases such as JNK, IKK, and Akt inhibits their protein activities [31]. Among those known phosphatases, protein tyrosine phosphatase (PTP) is highly vulnerable to this reversible oxidation [69,70]. PTPs, act in concert with protein tyrosine kinases to control essential cellular functions, have a highly conserved catalytic motif (I/V)HC(X_5_)R(S/T) that includes an invariant catalytic Cys residue [71]. This active site displays a low pK_a_ and renders Cys highly susceptible to oxidation [72]. At normal physiological condition, modest ROS production following agonist stimulation transiently oxidizes the Cys to the sulfenic acid (S-OH) form [69]. Only under severe oxidation can irreversibly convert this Cys to the sulfinic (S-O_2_H) or further to sulfonic (S-O_3_H) acid form [72]. ECs under laminar shear stress with modest ROS production may generate the reversible sulfenic acid form of PTPs and transiently inhibits PTP activity. Intriguingly, PTPs exposed to NO elicited a highly reversible enzyme inhibition via S-nitrosation (R-S-NO) [73,74]. Furthermore, cells treated with a low concentration of H_2_O_2_ leads to transient S-nitrosation of PTP [75]. PTP inactivation by S-nitrosation also contributes to an increase of insulin sensitivity in cells [76]. The activity of Src homology region 2-domain phosphatase-2 (SHP-2), a family member of PTPs, was shown to be inhibited by shear stress [77]. Oxidative or S-nitrosative modification of SHP-2 may be involved in this inhibition effect. Our studies demonstrated that ECs under steady laminar shear stress or in the presence of NO donor increased S-nitrosation of endothelial proteins [78,79]. As noted above, the catalytic Cys of PTP is vulnerable to oxidative modification, and thus there is likely a competition between S-nitrosation and oxidation on catalytic Cys of this PTP. We showed previously that a prior S-nitrosation protects PTP from irreversible oxidative modification and thus safeguard the activities of PTPs [73]. The highly reversible nature and the preemptive effect of S-nitrosation on PTPs by shear flow may be essential for modulating signaling responses during endothelial remodeling under shear stress, even in an inflammatory state (Figure 7).

### Effect of shear-induced ROS/NO on transcriptional factors

Earlier studies demonstrated that ROS generated by hemodynamic forces mediates the expression of *c-fos*[19], *egr-1*[80]. Previous studies have shown that disturbed flow leads to activation of transcription factors activation of activator protein 1 (AP-1) and nuclear factor kappa B (NFκB), whereas steady laminar flow lead to activation of Krüppel-like factor 2 (KLF2) and Nuclear factor (erythroid-derived 2)-like 2 (Nrf2) [65]. Our earlier study indicated that exposure of ECs to laminar shear stress of 12 dyn/cm^2^ induced Nrf2 nuclear translocation and this process was ROS-dependent [50]. An enhancement of the antioxidant response element (ARE)-binding activity of Nrf2 resulted in an increased expression of HO-1 [50]. These data indicate that the anti-atherogenic effect of steady laminar flow is at least partially due to the Nrf2 activation. The activation of transcription factor Nrf2 is also regulated by H_2_O_2_ and NO via the oxidation of Keap1 (a repressor of the Nrf2) at its critical cysteine residue, leading to Keap1 inactivation and thus allowing translocation of Nrf2 to the nucleus for initiating transcription of target genes [81]. Taken together, these findings support the concept of redox regulation of signaling pathways in flow-treated ECs, as proposed in Figure 6. However, there are still many inconsistencies among various studies on redox-responsive transcription systems, mainly due to the complexity and spatiotemporal factor of the redox-sensitive systems [82]. Fluid shear stress also induces transcriptional factors, such as KLF2, which upregulate eNOS expression [47-49]. Steady or PSS markedly activates Nrf2 and induces Nrf2-regulated antioxidant genes, such as heme oxygenase-1 and thioredoxin reductase-1 (TrxR1), and this reduces the level of intracellular O_2_^˙-^, thereby increasing the level of bioavailability NO [50-52].

### Effect of shear-induced ROS/NO on adhesion molecules and others

Earlier studies demonstrated that a modest increase of ROS mediated cyclic strain-induced expression of monocyte chemotactic protein-1 (MCP-1), intercellular adhesion molecule-1 (ICAM-1) [80,83] and shear stress-induced intercellular adhesion molecule-1 (ICAM-1) [56]. Oscillatory shear stress stimulated adhesion molecules (VCAM-1, ICAM-1 and E-selectin) expression in ECs and this upregulation could be suppressed in the presence of antioxidant (NAC), indicating oscillatory shear stress-induced signals are redox sensitive [84]. Shear stress increases ICAM-1 but decreases VCAM-1 and E-selectin expression induced by TNFα, indicating differential roles of shear stress in modulating TNFα-induced expression of adhesion molecules [85]. Using parallel-plate flow system, ECs cocultured with smooth muscle cells induced ICAM-1, VCAM-2 and E-selectin expression. However, these coculture effects are inhibited by shear stress [86]. High shear stress also suppressed tumor cell-ECs coculture-induced adhesion molecule expression [87]. To study the hemodynamic influence on the aortic valve inflammation, aortic surface of porcine aortic valve leaflets were exposed for 48 hours to pulsatile or oscillatory shear stress. Surprisingly, pulsatile shear stress, but not oscillatory shear stress, increased expression of the VCAM-1 and ICAM-1 [88]. In contrast, NO donor treatment reduced TNFα-induced VCAM-1 and ICAM-1 expression in ECs [89]. Indeed, shear flow increases NO-mediated S-nitrosation of proteins in ECs [78]. How this shear-induced S-nitrosative proteins modulating endothelial responses to cytokines remain to be determined. Structural proteins such as actin and integrin alpha6 have been shown to be S-nitrosated and thioredoxin reductase is responsible for actin denitrosation [90,91]. S-nitrosation of actin accelerates actin filament turnover and S-nitrosation of integrin alpha6 increases cancer cell migration [90,91]. It remains to be determined whether shear stress increases S-nitrosation of these structural proteins and modulates endothelial remodeling under flow conditions.

### Effect of shear-induced ROS/NO on protein-modification

Many Cys-containing proteins such as signaling molecules and transcriptional factors are potential targets that undergo a range of ROS-dependent or reactive nitrogen species (RNS)-dependent oxidative and nitrosative modifications of this Cys-containig proteins. Physiologically, NO through S-nitrosation of proteins regulates numerous cellular responses. NO exerts as an antioxidant by inhibiting NADPH oxidase activity via S-nitrosation [92]. NO was shown to promote the ROS scavenging activity of thioredoxin-1 via S-nitrosation on Cys69 residue [93,94]. Indeed, ECs under physiological shear stress increased protein S-nitrosylation [78,95] independent of cGMP-dependent signaling. In contrast, ECs with TNFα and mild oxidized low density lipoprotein (LDL) treatment reduced S-nitrosation [96]. Early researches demonstrated that AP-1 activity was altered by S-nitrosylation [97] and also by oxidation of Cys residues in AP-1 [98]. Furthermore, H_2_O_2_ treatment inhibited AP-1 activity and decreased eNOS promoter activity [99]. NFκB, AP-1, and p53 all contain reactive thiols in their DNA binding regions, the modification of which alters their binding to DNA. Thus, the dynamic interplay of ROS and NO and their oxidative and S-nitrosative modification of signaling molecules and/or regulatory protein thiols may be responsible for the consequent endothelial physiology under different flow conditions. The ROS and NO production rates in ECs under different flow patterns, leading to the differential activation/regulation of these thiol-proteins and thus results in anti-atherogenic (e.g. SOD, HO-1 expression) or pro-atherogenic effects (e.g. MCP-1, ICAM-1 expression) via different signaling pathways regulated by key transcription factors such as Nrf2, KLF2, AP-1, NFκB, etc.

### Effects of flow patterns on redox signaling and gene expressions

As mentioned earlier, the geometric structure of the vascular tree comprises straight, curved, branched, and many other complex features. *In vivo* evidence indicates that the atherosclerotic lesions preferentially localize at bends and bifurcations in the arterial tree with irregular flow patterns (disturbed with low and reciprocating (oscillatory) shear regions) [6]. However, no signs of atherosclerotic lesions appear in the straight part of the arterial tree where regular flow patterns (laminar with physiological shear stresses) predominate. Many studies have demonstrated that regular flow causes activation and regulation of anti-atherogenic and anti-inflammation genes, whereas irregular flow increases transcription of pro-atherogenic genes [1,63,65].

Based on available evidence and our previous discussion, the differential cellular response to different flow patterns may be explained by Figure 6: A regular flow pattern produces lower levels of ROS and higher NO bioavailability, leading to an anti-oxidative state and thus creating an anti-atherogenic environment via the expression of SOD, HO-1, etc. Conversely, an irregular flow pattern results in higher levels of ROS and yet lower NO bioavailability, giving rise to oxidative state and thus triggering pro-atherogenic effects through the expression of MCP-1, ICAM-1, etc. The irregular flow-induced low NO bioavailability is partly caused by the reaction of ROS with NO to form peroxynitrite, a key molecule which may initiate many pro-atherogenic events (Figure 6).

### Effect of shear stress on S-nitrosation

Increased NO production by eNOS activation in ECs under shear stress modulates various cellular processes that are essential for endothelial integrity. S-nitrosation involved in posttranslational regulation of many proteins that modulate cardiovascular function [14,100-103]. eNOS-derived NO selectively S-nitrosates many endothelial proteins and modulate diverse cell processes [104], including migration [105], permeability [106,107], oxidative stress [92,108], aging [109], and inflammation [110,111]. Current methods for detecting S-nitrosated proteins involve three key steps: 1) blocking free Cys thiols (−SH) by alkylation reagents [such as methyl methanethiosulfonate (MMTS) and iodoacetamide (IAM)] [101,112]. 2) Reduction of (S-NO) to free thiol (−SH) by ascorbate, and 3) free thiol is then labeled by biotin or CyDye (CyDye switch) [78,95,101]. After protein separation by two-dimensional gel electrophoresis (2-DE), the S-nitrosated proteins were subsequently analyzed and determined by LC-MS/MS. Using CyDye switch method coupled with two-dimensional gel electrophoresis, we demonstrated that shear induced eNOS activation in ECs led to S-nitrosation of more than one hundred proteins [78,79]. Several of which may be essential for endothelial remodeling. Interestingly, S-nitrosation may, by providing a negative feedback that limits eNOS activation, also affect vascular tone. S-nitrosation disrupts eNOS dimmers, leading to decreased eNOS activity [113,114]. This is supported by the fact that eNOS in resting cells is S-nitrosated; treatment with eNOS agonist vascular endothelial growth factor (VEGF) causes rapid denitrosation and eNOS activation although the mechanisms of S-nitrosation/denitrosation are unclear [115]. Furthermore, S-nitrosation of chaperone heat shock protein (Hsp90) suppresses its stimulatory effect on eNOS activity [116]. Thus, eNOS-derived NO production in ECs is regulated via the S-nitrosation/denitrosation of eNOS and eNOS dependent regulatory proteins, although the detailed control mechanisms are unclear.

We and others have shown that shear induces S-nitrosylation of endothelial proteins [78,95]. Presumably such changes drive vascular remodeling with flow. Shear stress-induced S-nitrosation is possibly dependent on the magnitude of the shear stress, consistent with the notion that endothelial NO production is proportional to the magnitude of the shear stress [78,95]. Importantly, eNOS-derived NO-mediated S-nitrosation is likely to be restricted to regions where eNOS are localized because higher concentration of NO is required to sustain protein S-nitrosation [104]. Intriguingly, in ECs treated with a NO donor (S-nitroso-N-acetylpenicillamine, SNAP) only a subset of the proteins became S-nitrosated [78]. This selective S-nitrosation in sheared ECs may be a consequence of a spatiotemporal partitioning of eNOS/NO and the vicinity of its target proteins within cellular compartments.

Recent studies indicate that protein S-nitrosation status in vivo is quite complex and involves a precisely regulated equilibrium between S-nitrosation and denitrosation reactions. These processes involve transnitrosation reactions between a variety of peptides and proteins. The consequent protein denitrosation can be critical in S-nitrosation-mediated signal mechanisms [117]. Whether shear flow and/or various flow patterns affect the equilibrium between S-nitrosation/denitrosation remains to be determined.

### Influence of shear stress on oxidative stress-induced inflammation of endothelium

ROS act as second messengers to transduce the shear signal and are thus important for the eventual physiological or pathophysiological response to shear. Under conditions of inflammation, the elevated ROS alter the NO/ROS equilibrium and the antioxidant status within cells. However, steady laminar flow exerts atheroprotection to ECs [1]. Indeed, laminar shear stress attenuates interleukin 6-induced JAK2/STAT3 activation [118] and interferon-γ-induced STAT1 activation in a shear magnitude-dependent manner [119]. Presumably, shear stress by suppressing elevations in ROS increases NO bioavailability and thus NO-mediated signaling, including S-nitrosation of regulatory proteins in ECs.

Although many S-nitrosated proteins have been identified in the cardiovascular system [120], and many such proteins are abundant and have been shown to be S-nitrosated in ECs under shear stress, the extent to which they may be protective is yet to be elucidated [95]. Studies have demonstrated S-NO-mediated suppression of NFκB-dependent expression of proinflammatory cytokines and adhesion molecules [107,118]. Overall, it is very likely that S-nitrosation of lower abundance signaling proteins play essential roles in atheroprotection. More targeted approach to identify S-nitrosated candidate proteins in ECs is needed. It is anticipated that the anti-inflammatory actions of NO via S-nitrosation is relevant across a range of vascular pathologies initiated by defective S-nitrosation. Since shear stress-induced activation of ECs is associated with S-nitrosation of many proteins, it is not surprising that an increase in flow and shear stress enhance eNOS expression and NO production play a crucial role in the prevention or retardation of progression of vascular diseases.

## Conclusions

It is now well established that low amounts of ROS are essential for normal cellular function, and increased ROS production contributes to vascular oxidative stress. ROS production varies under different flow patterns and conditions and this differential production modulates endothelial gene expression through complex mechanotransduction processes, to induce atheroprotective (laminar flow) or atherogenic (disturbed flow) endothelial phenotype and formation of an early atherosclerotic plaque. The redox regulation of shear signal also involves NO; indeed there is a very important role for ROS/NO interactions and S-nitrosation in mechano-signaling. The bioavailability of NO, S-nitrosation of transcription factors and other signaling proteins may be important determinants of vascular endothelial homeostasis under various flow conditions. The dynamic nature and consequences of oxidative and S-nitrosative proteins in sheared endothelial cells and its relevance to the atheroprotection are important topics for future studies.

## Abbreviations

AP-1: Activator protein-1; ARE: Antioxidant response element; ECs: Endothelial cells; eNOS: Endothelial cell NO synthase; FAK: Focal adhesion kinase; GPCR: G protein-coupled receptor; HO-1: Heme oxygenase-1; ICAM-1: Intracellular adhesion molecule-1; KLF2: Krüppel-like factor 2; LDL: Low density lipoprotein; MCP-1: Monocyte chemotactic protein-1; NF-kB: Nuclear factor kappa B; NO: Nitric oxide; Nox: NADPH oxidase; Nrf2: Nuclear factor (erythroid-derived 2)-like 2; OSS: Oscillatory shear stress; PSS: Pulsatile shear stress; PTP: Protein tyrosine phosphatase; RNS: Reactive nitrogen species; ROS: Reactive oxygen species; SHP-2: Src homology region 2-domain phosphatase-2; SOD: Superoxide dismutase; TrxR1: Thioredoxin reductase-1; VCAM-1: Vascular cell adhesion molecule-1; VEGF: Vascular endothelial growth factor; XO: Xanthine oxidase.

## Competing interests

The authors declare that they have no competing interests.

## Authors’ contributions

HJH and DLW collected information, organized and wrote this manuscript, and also designed conceptual figures. CAL, BH, and AHHT provided helpful suggestions and information. All authors read and approved the final manuscript.
